# Process Characterizations of Ultrasonic Extruded Weld-Riveting of AZ31B Magnesium Alloy to Carbon Fiber-Reinforced PA66

**DOI:** 10.3390/polym16121749

**Published:** 2024-06-20

**Authors:** Zeguang Liu, Guanxiong Lu, Yuanduo Yang, Sansan Ao, Kaifeng Wang, Yang Li

**Affiliations:** 1 School of Materials Science and Engineering, Tianjin University, Tianjin 300354, China; liuzeguang2021@163.com (Z.L.); yyd111865@tju.edu.cn (Y.Y.); ao33@tju.edu.cn (S.A.); 2 Quality Assurance Department, Volkswagen Automatic Transmission (Tianjin) Co., Ltd., Tianjin 300301, China; a04208222@163.com; 3 Key Laboratory of Mechanism Theory and Equipment Design of Ministry of Education, Tianjin University, Tianjin 300354, China; 4 International Institute for Innovative Design and Intelligent Manufacturing of Tianjin University in Zhejiang, Shaoxing 312000, China

**Keywords:** ultrasonic extruded weld-riveting, dissimilar joining, carbon fiber-reinforced thermoplastic, magnesium

## Abstract

Traditional metal–plastic dissimilar welding methods directly heat the metal workpiece, which may cause potential thermal damage to the metal workpiece. Ultrasonic extruded weld-riveting (UEWR) is a relatively new method for dissimilar joining of carbon fiber-reinforced thermoplastic (CFRTP) and metal. In this method, the CFRTP workpiece is melted using the ultrasonic effect and is squeezed into prefabricated holes in the metal workpiece to form a rivet structure. In this method, the metal workpiece is not directly heated, and potential high-temperature losses can be avoided. This paper investigates the process characterizations of UERW of AZ31B magnesium alloy to carbon fiber-reinforced PA66. The process parameters are optimized by the Taguchi method. The joint formation process is analyzed based on the fiber distribution in the cross-sections of joints. The effects of welding parameters on the joint microstructure and fracture surface morphology are discussed. The results show that a stepped amplitude strategy (40 μm amplitude in the first stage and 56 μm amplitude in the second stage) could balance the joint strength and joint appearance. Insufficient (welding energy < 2600 J or amplitude-A < 50%) or excessive (welding energy > 2800 J or amplitude-A > 50%) welding parameters lead to the formation of porous defects. Three fracture modes are identified according to the fracture surface analysis. The maximum tensile shear strength of joints at the optimal parameters is about 56.5 ± 6.2 MPa.

## 1. Introduction

The application of metal–thermoplastic hybrid structures contributes to the realization of lightweighting in aviation and aerospace, which promotes energy conservation and emission reduction [1]. Carbon fiber-reinforced thermoplastic (CFRTP) has attracted attention due to its advantages of low density, high specific strength, good corrosion resistance and potential for recycling [2]. In recent years, the joining between CFRTP and lightweight metals (aluminum alloy, magnesium alloy, etc.) has been extensively studied and used in actual industrial production.

Traditionally, mechanical joining (bolting, riveting, etc.) and adhesive bonding have been used for joining metal to carbon fiber composites (mainly carbon fiber-reinforced thermoset composites). However, the usage of fasteners requires holes drilled in the composites, which interrupts the continuity of the fibers. Additional fasteners also increase the mass of the whole structure [3,4]. For adhesive bonding, extra surface preparation process and long curing time are required. The joint is also sensitive to the environment [3,4]. For CFRTP, because of the weldability of its thermoplastic resin, welding is considered as a promising technology for joining metal/CFRTP, and, therefore, the issues of mechanical joining and adhesive bonding can be avoided. The mainstream method of metal/CFRTP welding is first to pre-treat the metal surface, such as by manufacturing macro- or microstructures on the metal surface. These structures help to increase the effective connection area and improve the mechanical interlocking between metal and CFRTP, as reported by Liu et al. [5]. Afterward, welding, such as laser welding, friction stir welding or hot-press welding, will be used to heat the metal sheet above the melting point of CFRTP. The CFRTP sheet is melted by the heat conduction of the metal, and the molten resin will be immersed into the macro- or microstructures on the surface of the metal sheet to form a joint. For example, Bi et al. [6] used a femtosecond laser to prepare micro- and nanostructures on the metal surface to enhance the joint strength of CF/PEEK to 6061 aluminum alloy. Kumar and Omkumar [7] dipped one end of a sandblasted aluminum sheet into molten PA11 and subsequently performed ultrasonic welding to join aluminum alloy to the polymer by micro-mechanical anchoring at the interface. Sarantinos et al. [8] machined different micro-pin structures on the metal surface for joining metal to CFRTP. Tan et al. [9] adopted micro-arc oxidation and silane coupling agent (SCA) treatment to modify the surface of a TC4 titanium alloy sheet and then welded it to CFRTP. Kim et al. [10] prepared special porous layers on a metal sheet and used a hydraulic hot press to join PA6 to 5052 aluminum alloy.

One problem with the above methods is that the metal sheet is directly heated, which may adversely affect the performance of the metal sheet, especially for metals with poor high-temperature resistance, such as magnesium alloys [11]. Recently, a low-heat input method based on ultrasonic welding was invented for dissimilar joining of metal to CFRTP by Li et al. [12] In this method, some prefabricated through-holes are machined on the metal sheet. The CFRTP sheet is placed on top of the metal one. Ultrasonic welding is used to direct melt the CFRTP sheets, and the molten CFRTP is squeezed into the prefabricated holes in the metal. A riveted metal/CFRTP joint will form after the cooling and solidification of the molten CFRTP. In this method, the metal sheet is not directly heated, and potential high-temperature losses can be avoided. This method is also metal-independent. Theoretically, it is suitable for all common metal materials. However, very few studies have been conducted on this method, and understanding of the process characterizations of this method is still unclear, which limits its application.

This paper names the above method “ultrasonic extruded weld-riveting (UEWR)”, and it is employed to join AZ31B Mg alloy to carbon fiber-reinforced PA66 (CF/PA66). Taguchi experiments are used to analyze the factors affecting the joint formation and tensile shear strength.

## 2. Experimental Procedures

### 2.1. Materials

AZ31B magnesium alloy and CF/PA66 sheets containing 40 wt% long fibers with dimensions of 100 × 40 × 2 mm^3^ and 100 × 40 × 3 mm^3^ were used in the experiments. The CF/PA66 sheets were injection-molded by pellets provided by EMS-CHEMIE (Suzhou, China) Ltd. (Grivory^®^ GCL-4H). The injection molding parameters were provided by the material supplier and are given in Table 1. The physical properties of the AZ31B and CF/PA66 are listed in Table 2.

**Figure 1:**
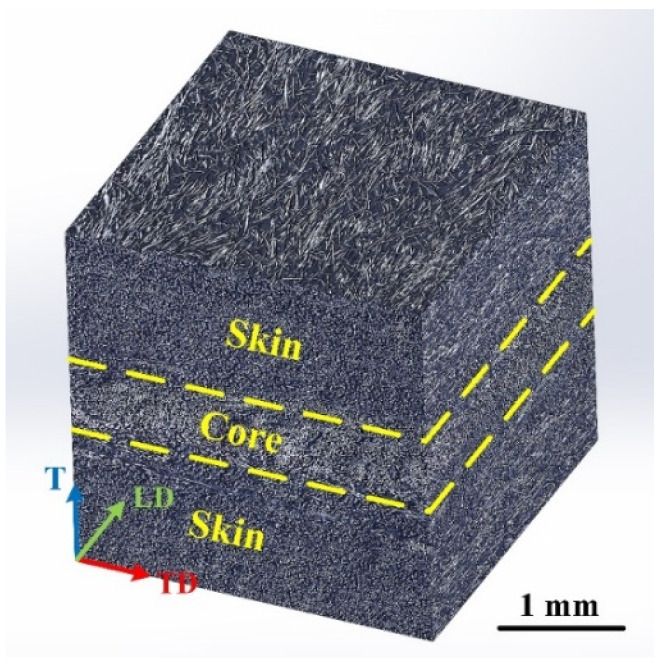
Three-dimensional display of the microstructure of the CF/PA66 sheet. “LD” indicates length direction (parallel with the injection direction), “TD” indicates transversal direction, and “T” indicates thickness direction.

The microstructure of the CF/PA66 in three dimensions is shown in Figure 1. The black part is the polymer matrix, and the white part is the carbon fiber. A clear “skin–core–skin” structure can be observed. The thicknesses of the “core” and “skin” layers were approximately 0.70 mm and 1.15 mm, respectively. The orientation of fibers in the “skin” layer is parallel to the length direction (LD) or injection direction of the sheet.

### 2.2. Ultrasonic Extruded Weld-Riveting

A 2000XD ultrasonic welder (produced by Branson Ultrasonics (Shanghai, China). Co., Ltd.) was used in the experiments. The welder is able to control the parameters of welding energy, amplitude, welding force, trigger force, etc. The maximum peak-to-peak amplitude (the set parameter value is 100%) of this welder is about 80 μm. Energy mode (welding stops when the output energy reaches the set value) was adopted in the experiments. An aluminum alloy sonotrode with a diameter of 20 mm was used.

**Figure 2:**
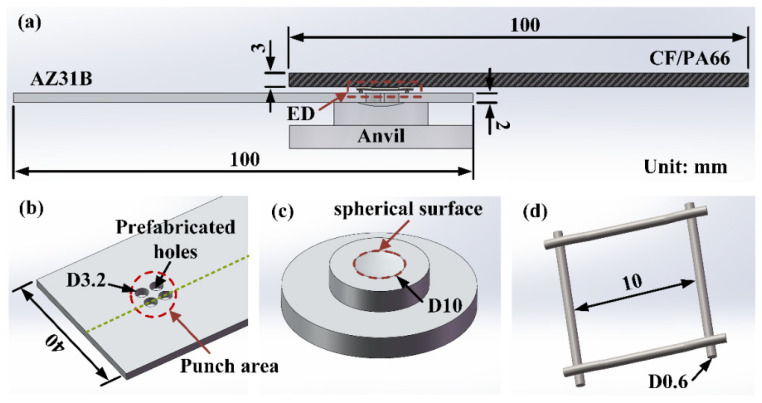
Schematic diagram of ultrasonic extruded weld-riveting process. (**a**) Schematic diagram of lap joint; (**b**) A magnesium alloy sheet with four prefabricated holes; (**c**) Anvil with a spherical concave zone; (**d**) A gird-type energy director.

As shown in Figure 2a, a CF/PA66 sheet (upper workpiece) was in direct contact with the sonotrode, and a magnesium alloy sheet (lower workpiece) was placed on the anvil; both of them were fixed by a clamping system. Four through-holes with a diameter of 3.2 mm were prefabricated at a certain position on the magnesium alloy sheet and distributed in a circular area (punch area) with a diameter of 10 mm, as shown in Figure 2b. The size and distribution of the through-holes were determined based on our preliminary study that the more holes there are, the better the joint strength will be for the same total volume of holes punched. The quantity of prefabricated holes decided on in this work is a balance between joint performance and pre-treatment workload. The schematic diagram of the anvil is shown in Figure 2c. A spherical concave area with curvature radius of 16 mm and diameter of 10 mm was fabricated on the surface of the anvil. The concave area was mainly designed to improve the bearing capacity of the joint in the direction of thickness. Since the viscoelastic heat generation of CF/PA66 is in direct proportion to its internal strain in the ultrasonic welding process, a commercially available grid-type energy director (ED) (10 × 10 mm^2^) made of stainless steel was used to increase the strain at the interface and promote more heat generation. Figure 2d shows the grid-type ED. The size of the ED is determined by the size of the sonotrode and the punch area, which has to be located within the welding area and not hinder the resin flow. Before welding, the ED was slightly embedded in the CF/PA66 surface to be welded by a hand-held ultrasonic welder to prevent it from moving during the welding.

**Figure 3:**
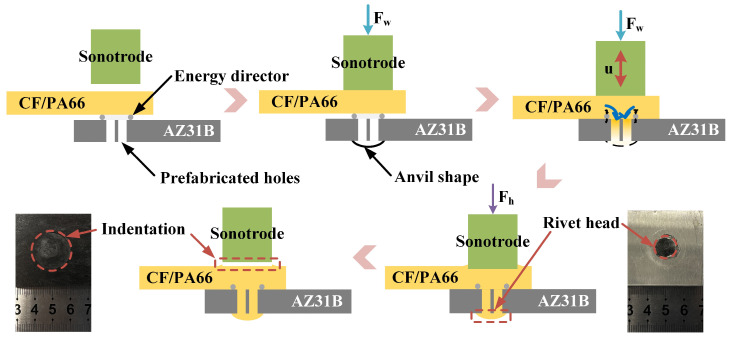
Schematic diagram of the ultrasonic extruded weld-riveting process. “Fw” means the welding force; “Fh” means the holding force.

The UEWR process is shown in Figure 3. The cutting plane is shown in Figure 2b as a green dashed line. At the beginning, the sonotrode contacted and applied force to the CF/PA66 sheet. When the force reached the preset trigger force during the loading process, the sonotrode started to vibrate perpendicular to the workpieces at a high frequency (20 kHz), and the force subsequently increased to the preset welding force. Under the combined action of friction heat, viscoelastic heat and the welding force, the polymer matrix melted rapidly and flowed into the prefabricated holes in the metal sheet and the spherical concave area on the anvil. The vibration stopped when the output energy of the welder reached the preset welding energy and the sonotrode applied a holding force to the workpiece for a holding time. After that, the sonotrode was raised and the UEWR was completed. The joining mechanism of this method is based on the macro-mechanical interlocking formed by a rivet structure. It is worth noting that due to the flow of the polymer matrix into to the prefabricated holes, the sonotrode will be pressed into the composite at some depth to form an indentation. This depth is approximately equal to the total volume of the four holes (4 × 3.14 × 1.6^2^ × 2 ≈ 64.31 mm^3^) divided by the tip area of the sonotrode (3.14 × 10^2^ = 314 mm^2^), which is approximately 0.2 mm. According to the experimental results, indentation of this depth did not adversely affect the mechanical property of the joint. Therefore, in this research, the effect of the indentation was ignored. But the effect of indentation depth on the joint mechanical property is an interesting topic, and it will be considered in a further study.

Based on the preliminary study, it was found that it was fairly easy for the composite to fill the prefabricated holes but difficult for it to enter the rivet head (the concave area on the anvil). This is due to the decrease of heat generation in the material system at the end of welding. It was found that using stepped amplitude, that is, dividing the welding process into two stages with a percentage of total welding energy as the threshold ratio, in which the amplitude in the second stage (amplitude-B) is higher than that in the first stage (amplitude-A), can effectively increase the heat generation in the later stage of welding, so that the molten CFRTP can be better flowed into the rivet head. Therefore, the total welding energy, threshold ratio of welding energy, amplitude-A and amplitude-B are considered as four factors affecting the weld quality. The other two parameters are welding force and trigger force. The ranges for the above parameters were determined by preliminary experiments. Then, Taguchi experiments with six factors and four levels were used to analyze the effects of ultrasonic welding parameters on the formation and mechanical property of joints. Taguchi experimental design is a fast and efficient design method for studying multifactorial and multilevel problems, which can utilize a limited number of trials to infer correct conclusions and obtain better results [13]. The Taguchi experiment design is shown in Table 3. The holding force was the same as the welding force, with a holding time of 3 s. Range analysis was conducted using “Minitab 21.2.0” software to obtain the optimum parameters and significantly influenced factors. The detailed calculation of the range analysis can be found in Refs. [14,15].

### 2.3. Tensile Shear Test and Microstructural Observation

The tensile shear test is conducted by using a universal testing machine under a crosshead speed of 2 mm/min. During the tensile shear test, two tensile grips clamped the two ends of the welded sample. Before clamping, two shims or spacers were added on both sides of the workpiece to maintain consistent thickness at both ends, thereby reducing the impact of joint rotation. Tensile shear strength (MPa) is calculated by dividing the tensile shear force (N) by the total area of the four holes (4 × 3.14 × 1.6^2^ ≈ 32.15 mm^2^). Each set of parameters is tested three times, and the results are averaged. The cross-sections and fracture morphologies of the joints were observed using a Smart Zoom5 microscope and SU1510 tungsten filament scanning electron microscope (SEM).

## 3. Results and Discussion

### 3.1. Weld Quality

**Figure 4:**
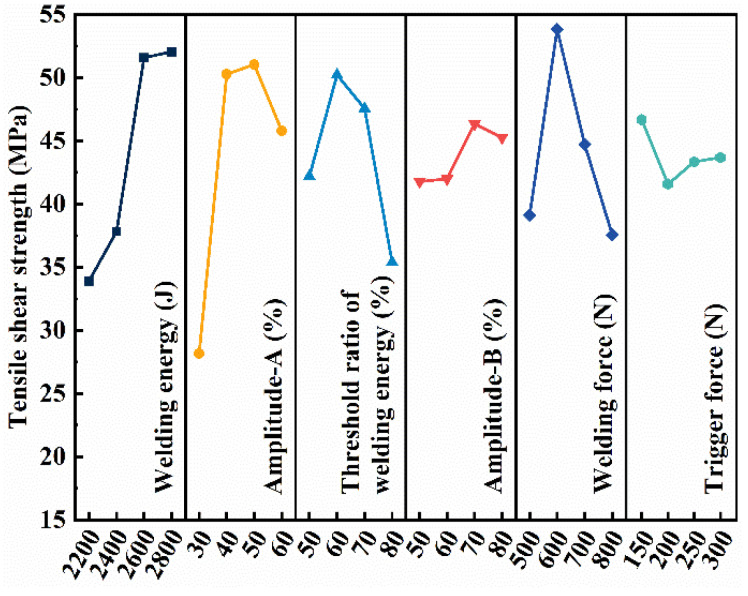
Main effect plot of process parameters on the mechanical property of the joint.

Figure 4 shows the effect of welding parameters on the tensile shear strength of the joint, where the value of the vertical coordinate (K*i*) is the combined arithmetic mean of the tensile shear strength obtained for the parameters indicated by the corresponding horizontal coordinate (*i*). The range R (max(Ki)–min(Ki)) reflects the degree of effect of each factor and level on the tensile shear strength. The larger the R value, the greater the degree of the effect of this factor on the tensile shear strength. The impact of parameters on the tensile shear strength in a descending order is amplitude-A, welding energy, welding force, threshold ratio of welding energy, amplitude-B and trigger force. The optimal parameters based on the mechanical property are listed in Table 4.

**Figure 5:**
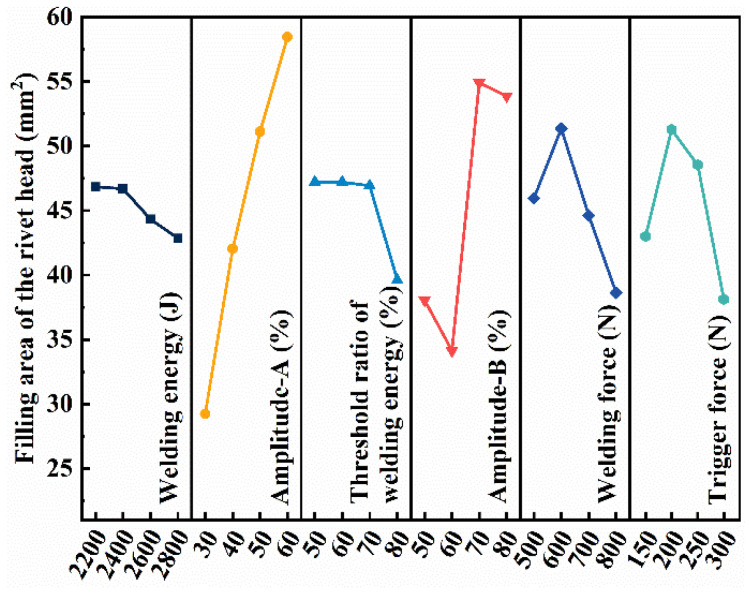
Main effect plot of process parameters on the filling performance.

In addition to the mechanical property, the filling of molten CFRTP into the prefabricated holes and the concave area on the anvil is another indicator reflecting the weld quality. In this study, filling performance is characterized by the projected area of the composite rivet head on the magnesium alloy sheet, which is measured by ImageJ (version 1.53p). Figure 5 shows the effect of welding parameters on the filling ability of molten CFRTP. The effect of the parameters on the filling ability in descending order is amplitude-A, amplitude-B, welding force, trigger force, threshold ratio of welding energy, and welding energy. The optimal parameters based on filling performance are also shown in Table 4.

Combining Figure 4 and Figure 5, welding energy has the most significant effect on the mechanical property and has little effect on filling performance. Amplitude-A has a significant effect on both the joint strength and filling ability, but amplitude-B has a greater effect on the filling ability. The threshold ratio of welding energy has greater impact on the joint strength. The optimized welding forces for the mechanical property and filling performance are the same. The trigger force plays a more important role on filling performance than on the mechanical property. To balance the joint strength and joint formation, the optimized welding parameters are listed in the “Balanced” row in Table 4.

**Figure 6:**
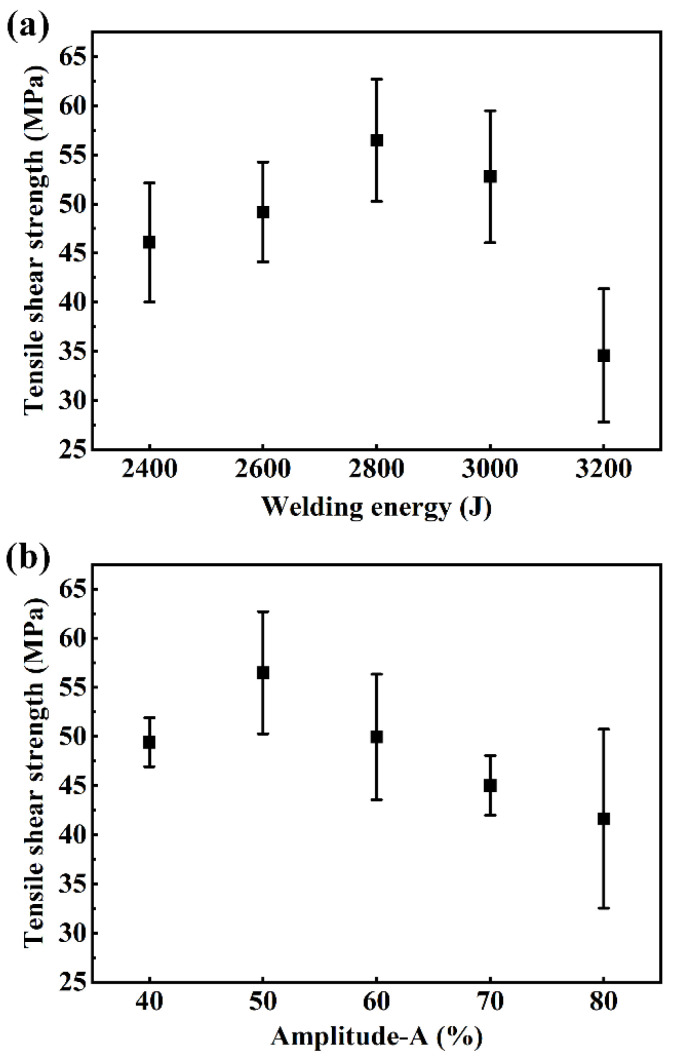
Tensile shear strength with a single variable. (**a**) Welding energy as the variable while amplitude-A is fixed at 50%; (**b**) Amplitude-A as the variable while the welding energy is fixed at 2800 J.

Since the welding energy and amplitude-A have the greatest impact on the weld quality, the single-variable method was used to make the energy (2400 J, 2600 J, 2800 J, 3000 J and 3200 J) or amplitude-A (40%, 50%, 60%, 70% and 80%) as a univariate to carry out further studies, and the other variables were the “Balanced” parameters shown in Table 3. The tensile shear strength with a univariate condition is shown in Figure 6. As shown in Figure 6a, the tensile shear strength increases first and then decreases with increasing welding energy. Similarly, in Figure 6b, the tensile shear strength increases first and then decreases with the rise of amplitude-A. When amplitude-A reaches 80%, the standard deviation increases significantly, and the strength of the joint becomes unstable. The joint strength obtained by UEWR reached 56.5 ± 6.2 MPa at the optimum parameters, i.e., welding energy of 2800 J and amplitude-A of 50%.

Table 5 lists the strengths of metal/CFRTP joints obtained by different welding or welding and riveting hybrid joining methods. The tensile shear strength of most welded joints was only about 5–10 MPa. The use of a welding and riveting hybrid joining method could increase the joint strength. For example, friction self-riveting welding can increase the joint strength to 27 MPa. Laser welding–riveting can increase the joint strength up to 10 kN depending on the material combination and process parameters. The joint strength of the present method reached 56.5 ± 6.2 MPa at the optimum parameters. Welding and riveting hybrid joining can give full play to the advantages of riveting and welding; therefore, the joint strength is higher than that of welding methods.

### 3.2. Cross-Sections of Joints

**Figure 7:**
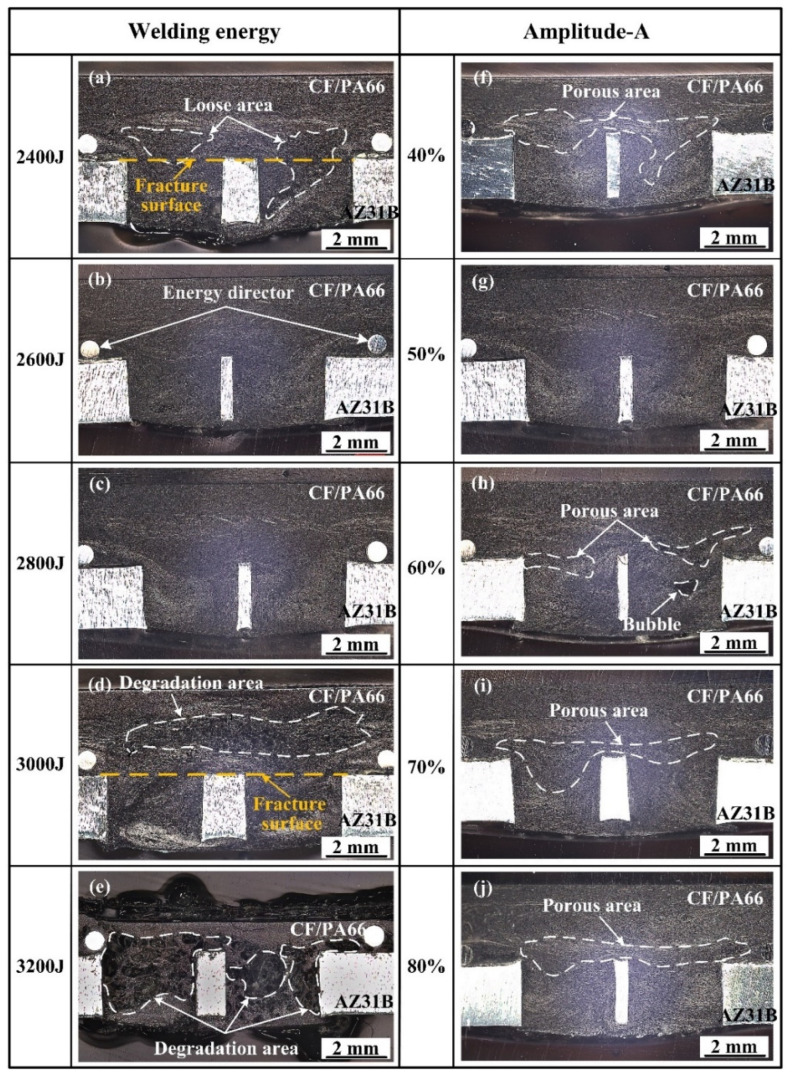
Cross-sectional microstructure at different parameters: (**a**–**e**) welding energies of 2400–3200 J while amplitude-A is fixed at 50%; (**f**–**j**) amplitude-A of 40%–80% when the welding energy is fixed at 2800 J.

**Figure 8:**
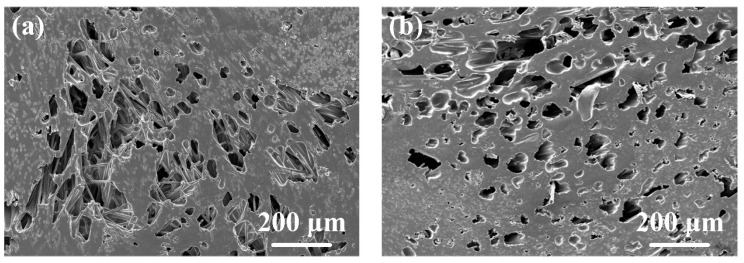
The typical morphologies of different defective areas. (**a**) loose area at 2400 J, 50% amplitude-A; (**b**) degradation area at 3000 J, 50% amplitude-A.

Figure 7 shows the cross-sections of the joints with different welding parameters. It was found that a loose area with a large amount of tiny voids would appear in the center of the joint, i.e., the area marked by the dashed line in Figure 7a, when the welding energy was 2400 J. An enlarged view of this area is shown in Figure 8a. In this case, the welding energy was not sufficient for the polymer matrix to degrade, so these voids were not caused by degradation. This is also evidenced by the irregular shape of the voids. In addition, as shown in Figure 1, there was no void defect in the polymer matrix of the composite. Hence, it can be inferred that these voids were caused by the insufficiency of filling. That is why this area is called a loose area.

When the welding energy reached 2600 J and 2800 J, the joints were well filled and had no obvious defect. When the energy reached 3000 J, the polymer matrix degraded to form a degradation zone, i.e., the area marked by the dashed line in Figure 7d. Figure 8b shows the magnification of this area. It can be seen that the voids caused by degradation are larger and smoother than those caused by poor filling. When the energy reached 3200 J, the polymer matrix underwent severe degradation, and a large number of voids appeared in the joint. Furthermore, the thickness of the CF/PA66 sheet in the welded area was severely reduced as the polymer matrix degraded and there was significant flow out of the weld area at 3200 J.

**Figure 9:**
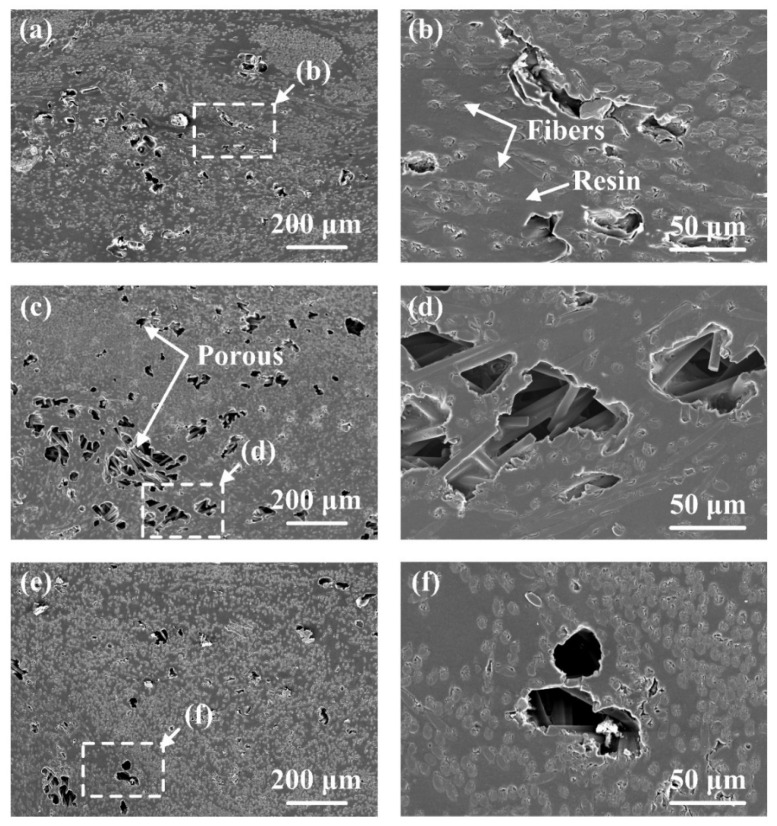
The typical morphologies of porous areas. (**a**,**b**) 2800 J, 40% amplitude-A; (**c**,**d**) 2800 J, 60% amplitude-A; (**e**,**f**) 2800 J, 70% amplitude-A.

Figure 7f–j show the effect of amplitude-A on the joint formation. It can be seen that porous areas appeared in the joints at different amplitude-A values except the 50% value of amplitude-A. The detailed morphologies of these porous areas are shown in Figure 9. Among them, Figure 9b, Figure 9d, Figure 9f are enlarged views of Figure 9a, Figure 9c and Figure 9e, respectively. Defects of similar size are observed in Figure 9a,c,e, indicating that the formation of defects is not much affected by amplitude-A but mainly by energy. Comparing Figure 9b,d,f, it is found that the contact between the fibers and the resin is not strong when amplitude-A is 40%, which may affect the material properties, while this phenomenon is improved when amplitude-A reaches 60%. Moreover, the void shape in Figure 9b is irregular and appears to be closer to that with poor filling, while the void shape in Figure 9f is close to circular, which is similar to the voids that appear in the degraded polymer matrix. The SEM fractography in the following texts also proves that the voids in Figure 9b are caused by insufficient filling, while the voids in Figure 9f are caused by degradation.

**Figure 10:**
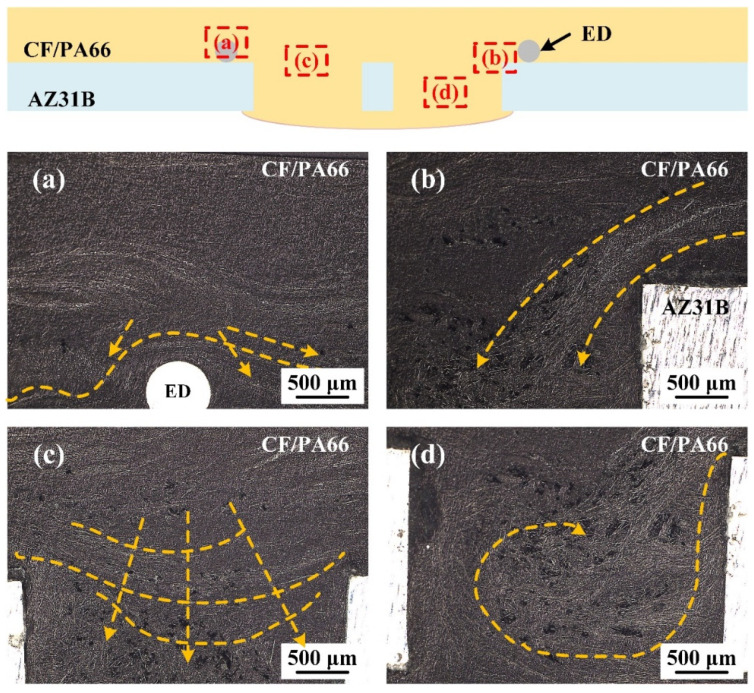
The typical distribution of carbon fibers in the locations (**a**–**d**) of a joint when the welding energy is 2600 J and amplitude-A is 50%.

**Figure 11:**
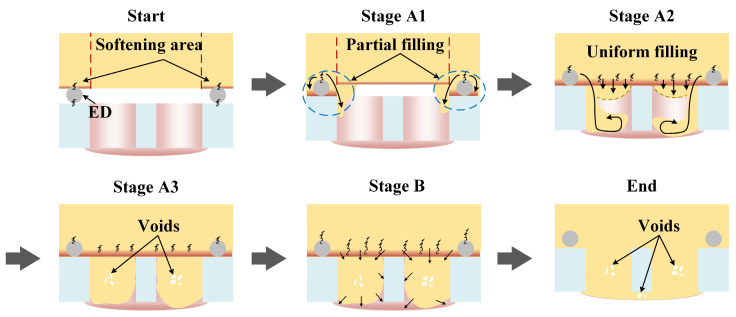
The hole-filling process in UEWR.

Figure 10 shows the typical distribution of carbon fibers in a joint, with the yellow arrows representing the flow direction of the composite. According to Figure 10a,b, it is found that the composite near the ED flowed along the sidewall into the prefabricated holes during the welding process. In Figure 10c, the composite in the middle of the prefabricated hole is shown to flow downward in an arc shape. Meanwhile, it is found from Figure 10d that composite backflow occurred in the lower part of the hole. The hole-filling process of the composite can be deduced from the fiber distribution, as shown in Figure 11. With amplitude-A vibration, since more heat is generated in the region where the ED is located, the polymer matrix in this zone is more prone to softening and melting. At the beginning, the ED is pressed into the softened polymer matrix due to heat and welding force. At stage A1, only the polymer matrix in the area where the ED is located melts first and flows into the prefabricated holes. In stage A2, all the polymer matrix in the joint area melts and flows. The composite near the ED continues to flow along the sidewall into the prefabricated holes and then forms a reflux when it reaches the bottom of the hole, while the composite in the other part of the hole flows into the hole forming an arc-shaped surface. Until stage A3, the prefabricated holes are basically filled with composite, and defects are easily formed in the center of the prefabricated holes as it is filled last. Furthermore, when the energy is relatively low, it is understandable that there is less uniform filling and more significant partial filling. More material will fill the holes along the sidewalls with less flow during uniform filling, which leads to defects close to the interface; when the energy is appropriate, the effect of uniform filling is balanced with partial filling, and this results in fewer defects. It was explained in Section 3.1 that the filling of the rivet head is mainly influenced by amplitude-B. Therefore, we can assume that the greater amplitude-B promotes the fluidity of the composite, leading to the formation of the rivet head in stage B.

### 3.3. Fractography

**Figure 12:**
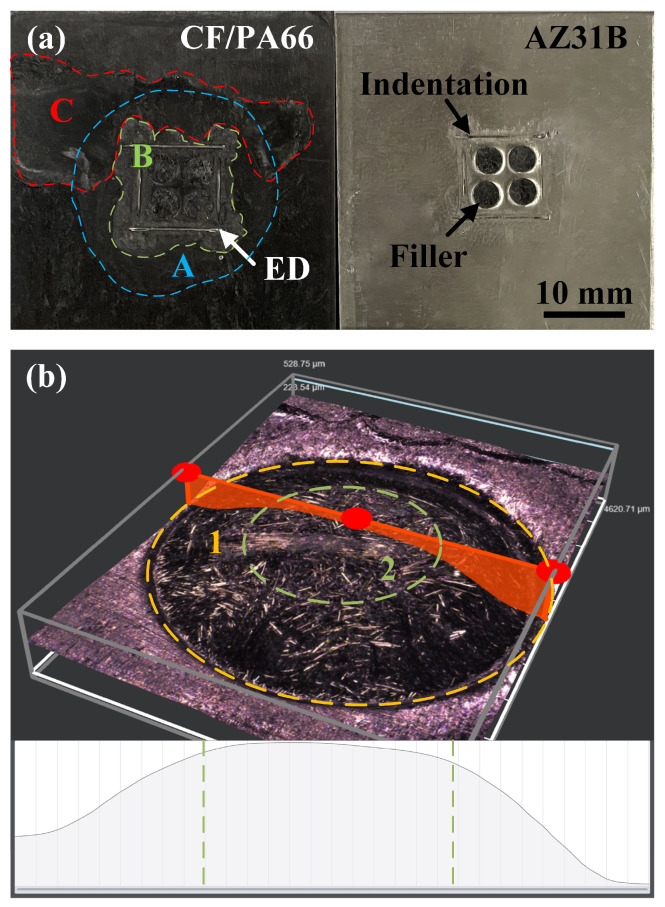
Typical fracture surfaces of a joint: (**a**) macroscopic view; (**b**)magnified contour of a single-hole fracture surface.

Figure 12 shows typical macroscopic fracture surfaces of a joint. The fracture occurs mainly at the interface of the joint, as shown in Figure 12a. On the metal side, the prefabricated holes were filled with the composite, and the filler remains in the holes after the fracture. The ED sank into the composite matrix due to the combined effort of welding force and interfacial heat and left an indentation on the metal surface. On the composite side, three different states of the melted polymer were observed. Region A, the area inside the blue dashed line, is directly below the sonotrode, where the polymer softened but did not flow or interact with the metal. Region B, the green area, is where the composite matrix melted due to the effect of the ED and filled the prefabricated hole. Region C is caused by the extrusion of the melted polymer in Region B. Figure 12b shows the three-dimensional morphology of a single-hole fracture surface on the composite side. The fracture surface was not evenly flat. The relatively deep dent in Region 1 is caused by the uneven edge of the hole during punching. Region 2 is flatter and has a uniform shape. Observation of Region 2 using SEM reveals significant differences in various ultrasonic welding parameters of the typical fracture morphology.

**Figure 13:**
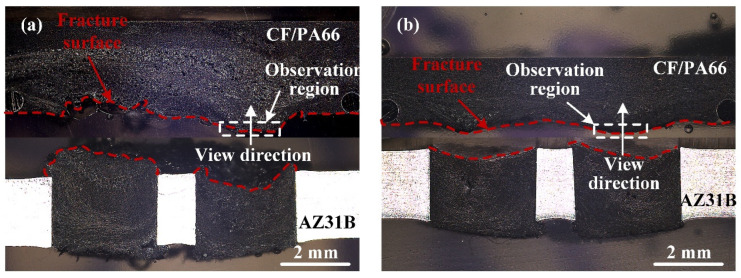
Interfacial fracture modes of typical joints. (**a**) 2400 J, 50% amplitude-A; (**b**) 2800 J, 50% amplitude-A.

Figure 13 illustrates two different interfacial fracture modes. When obvious defects exist at the interface, the crack will propagate along these defects, and the fracture surface profile is irregular and influenced by the shape of the defects, as shown in Figure 13a. The other fracture is an arc-shaped fracture, and the formation of an arc-shaped surface is related to the fiber distribution. This fracture mode occurs when there is no obvious defect at the interface, as shown in Figure 13b.

**Figure 14:**
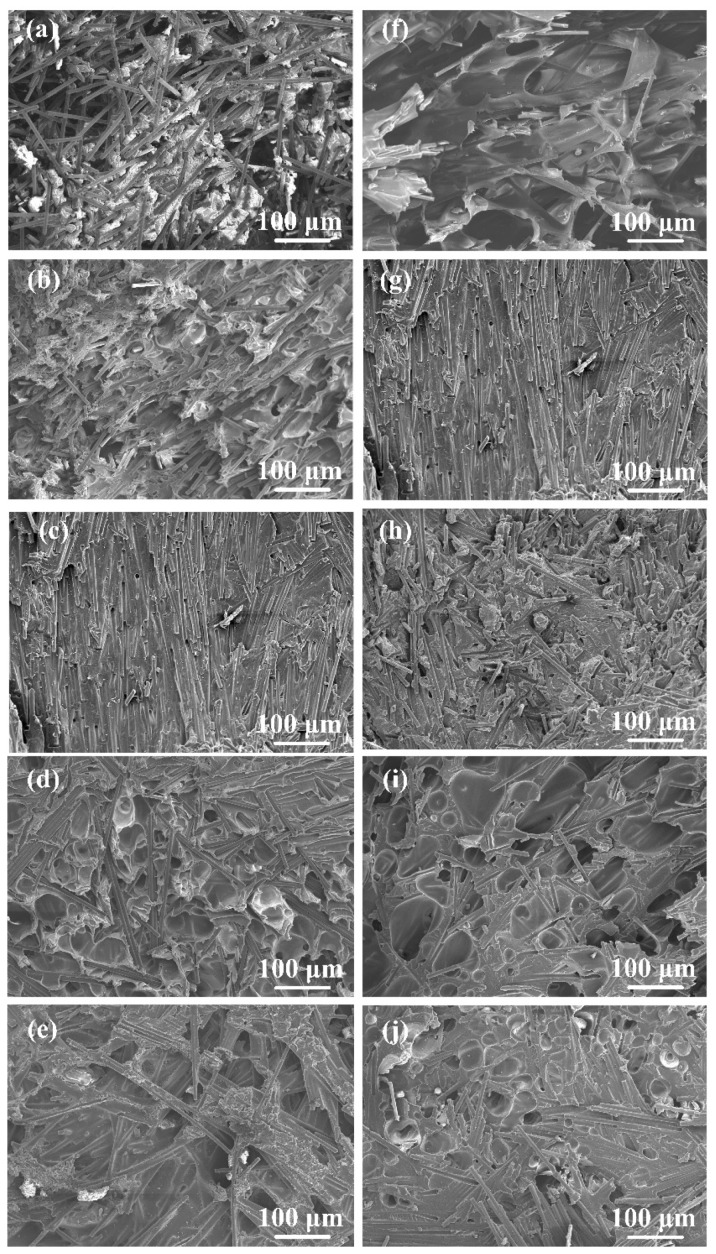
Fracture surface morphologies at various welding parameters: (**a**) 2400 J, 50% amplitude-A; (**b**) 2600 J, 50% amplitude-A; (**c**) 2800 J, 50% amplitude-A; (**d**) 3000 J, 50% amplitude-A; (**e**) 3200 J, 50% amplitude-A; (**f**) 2800 J, 40% amplitude-A; (**g**) 2800 J, 50% amplitude-A; (**h**) 2800 J, 60% amplitude-A; (**i**) 2800 J, 70% amplitude-A; (**j**) 2800 J, 80% amplitude-A.

**Figure 15:**
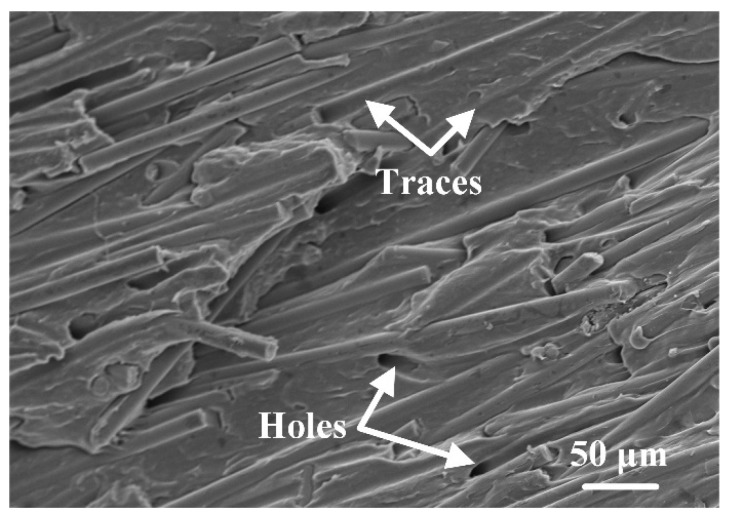
Fiber–matrix debonding fracture surface morphology.

Figure 14 shows the morphologies of fracture surfaces with different welding parameters. The location of the observation regions in Figure 14 is marked in Figure 13. Figure 14a–e show the effect of welding energy on the fracture surface morphology. When the welding energy was 2400 J (Figure 14a), there was some space among fibers that was not filled by the polymer matrix, indicating the filling was not solid. As the welding energy increased, the filling effect improved, and the size and number of defects at the interface decreased significantly. The fracture surfaces were relatively flat, and the fibers were ordered in a uniform direction nearly parallel to the stretching direction, as shown in Figure 14b,c. Additionally, traces and holes were found on the fracture surface after the fibers were pulled out of the polymer matrix. The failure mechanism is considered as fiber–matrix debonding, as shown in Figure 15. When the welding energy was higher than 2800 J, as shown in Figure 14d,e, the fiber distribution in the fracture surface became chaotic, and many circular dimples (30–50 μm in diameter) and elliptical dimples (about 100–150 μm on the long axis and 30–80 μm on the short axis) can be observed, indicating the degradation of the polymer matrix.

Similar phenomenon can be found in Figure 14f–j. When amplitude-A was 40%, as shown in Figure 14f, some space among fibers could be observed, verifying that the porous area in Figure 7f and Figure 9a,b was caused by insufficient filling. Compared with 50% amplitude-A, the fiber distribution in the fracture surface of 60% amplitude-A was more chaotic, as shown in Figure 14h. This indicates that the defects in the joint began to increase under this parameter, but, in general, the failure mechanism of the joint was still fiber–matrix debonding. These defects in the 60% amplitude-A joint were relatively few. When amplitude-A reached 70% and 80%, as shown in Figure 14i,j, obvious smooth voids could be observed, indicating the polymer matrix experienced degradation. The porous areas in Figure 7i,j were caused by degradation.

In summary, when the welding energy or amplitude-A was insufficient or excessive, the joints fractured along internal defects. When the welding parameters were suitably selected, no obvious defect could be observed in the joint, and the joint failed through fiber–matrix debonding.

## 4. Conclusions

This paper investigates the ultrasonic extruded weld-riveting of AZ31B sheets to CF/PA66 sheets. The main conclusions that can be drawn are as follows:

(1) Welding energy and amplitude-A have greater impact on joint strength. A stepped amplitude strategy could promote the flow of molten resin and facilitate better joint appearance. The maximum tensile shear strength is about 56.5 ± 6.2 MPa with the optimal parameters (welding energy 2800 J, amplitude-A 50%, threshold ratio of welding energy 60%, amplitude-B 70%, welding force 600 N and trigger force 200 N).
(2) Two flow patterns of molten resin are observed. One is when the molten resin flows along the sidewall of the holes and refluxes when it reaches the bottom of the holes, and the other is when the molten resin flows into the hole uniformly in an arc shape.
(3) Insufficient (welding energy < 2600 J or amplitude-A < 50%) or excessive (welding energy > 2800 J or amplitude-A > 50%) heat input will introduce porous areas inside the joint, causing the joints fracture along the porous areas. With optimal welding parameters, the joints fail through fiber–matrix debonding.

## Figures and Tables

**Table 1:** The injection molding parameters.

Parameter	Value
Feed temperature (°C)	80
Zone 1 temperature (°C)	290
Zone 2 temperature (°C)	300
Zone 3 temperature (°C)	310
Nozzle temperature (°C)	300
Tool temperature (°C)	100
Hold-on pressure (bar)	550
Dynamic pressure (bar)	40
Screw speed (m/min)	7

**Table 2:** The thermal and mechanical properties of the AZ31B and CF/PA66 sheets.

Materials	Glass Transition Temperature (°C)	Melting Point (°C)	Density (g/cm^3^)	Tensile Modulus (GPa)	Tensile Strength (MPa)	Elongation at Break (%)
AZ31B	---	650	1.78	45	260	9
CF/PA66	85	260	1.34	29.5	335	1.4

**Table 3:** Taguchi design of UEWR parameters.

Parameters	Levels			
	1	2	3	4
Welding energy (J)	2200	2400	2600	2800
Amplitude-A (%)	30	40	50	60
Threshold ratio of welding energy (%)	50	60	70	80
Amplitude-B (%)	50	60	70	80
Welding force (N)	500	600	700	800
Trigger force (N)	150	200	250	300

**Table 4:** Optimized welding parameters.

	Welding Energy (J)	Amplitude-A (%)	Threshold Ratio of Welding Energy (%)	Amplitude-B (%)	Welding Force (N)	Trigger Force (N)
Mechanical property	2800	50	60	70	600	150
Filling performance	2200	60	50	70	600	200
Balanced	2800	50	60	70	600	200

**Table 5:** Comparison of joint strength manufactured by different welding or welding and riveting hybrid joining methods with varied pre-/post- treatments.

Methods	Materials	Pre-Treatment/Post-Treatment	Tensile Shear Strength/Force	Ref.
Ultrasonic welding	AZX612/CF-PA6	Plasma electrolytic oxidation treatment of Mgalloy surface and preheating before welding/None	5.7 MPa	[16]
Hot-pressing joining	AZ31B/CF-PEEK	Laser texturing of mg alloy surface/None	5.4 MPa *	[17]
Hot-pressing joining	AZ31/CF-TPU(CF/PA66)	Alcohol cleaning of workpiece surface/Annealing	4.6 MPa * for AZ31/CF-TPU; 6.9 MPa * for AZ31/CF/PA66	[18]
Hot-pressing joining	AZ31/CF-TPU	None/Annealing	4.6 MPa *	[19]
Laser welding	AZ31/PU	Alcohol cleaning of workpiece surface/Annealing	4 MPa *	[20]
Laser welding	AZ91D/PET	Laser melting treatment of Mg alloy surface/None	8.7 MPa	[21]
Laser welding	AZ31B/CF-PEEK	Polishing the oxide layer on the AZ31B surface with abrasive papers and ultrasonic cleaning of the workpieces/None	9 MPa	[22]
Laser welding	AZ31/CF/PA66	Micro-arc oxidation treatment/None	13.3 MPa	[23]
Laser-TIG welding–riveting	AZ31B/CF-PEEK	Punching, specially designed rivet	1.4 kN	[24]
Laser welding–riveting	TC4/CF-PEEK	Punching, specially designed rivet	8.5 kN	[25]
Laser welding–riveting	AA6061/CF-PEEK	Punching, specially designed rivet	4.7 kN	[26]
Laser welding–riveting	DP980/CF-PEEK	Punching, specially designed rivet	10 kN	[27]
Friction welding	AZ31B/PA6	Gritting the Mg alloy surface with #800 sandpaper/None	5.8 MPa *	[28]
Friction self-riveting welding	AA2060/GF-PPS	Prefabricated holes in Al sheet/None	27 MPa	[29]

* The value is calculated by the authors according to the force and weld area in the reference.

## Data Availability

Data are contained within the article.
